# Bioequivalence Methodologies for Topical Drug Products: ***In Vitro*** and ***Ex Vivo*** Studies with a Corticosteroid and an Anti-Fungal Drug

**DOI:** 10.1007/s11095-017-2099-1

**Published:** 2017-01-17

**Authors:** Leila Bastos Leal, Sarah F. Cordery, M. Begoña Delgado-Charro, Annette L. Bunge, Richard H. Guy

**Affiliations:** 1 Department of Pharmacy & Pharmacology, University of Bath, Claverton Down, Bath, BA2 7AY UK; 2 Departamento de Ciências Farmacêuticas, Universidade Federal de Pernambuco, CEP: 50740-520 Recife-PE, Brazil; 3 Chemical and Biological Engineering Department, Colorado School of Mines, Golden, Colorado 80401 USA

**Keywords:** dermatopharmacokinetics, *in vitro* release test, *in vitro* skin penetration, IVIVC, topical bioequivalence

## Abstract

**Objective:**

To examine whether *in vitro* and *ex vivo* measurements of topical drug product performance correlate with *in vivo* outcomes, such that more efficient experimental approaches can be reliably and reproducibly used to establish (in)equivalence between formulations for skin application.

**Materials and Methods:**

*In vitro* drug release through artificial membranes, and drug penetration into porcine skin *ex vivo*, were compared with published human *in vivo* studies. Two betamethasone valerate (BMV) formulations, and three marketed econazole nitrate (EN) creams were assessed.

**Results:**

For BMV, the stratum corneum (SC) uptake of drug in 6 h closely matched data observed *in vivo* in humans, and distinguished between inequivalent formulations. SC uptake of EN from the 3 creams mirrored the *in vivo* equivalence in man (both clinically and *via* similar tape-stripping experiments). However, EN clearance from SC *ex vivo* did not parallel that *in vivo*, presumably due to the absence of a functioning microcirculation. *In vitro* release of BMV from the different formulations did not overlap with either *ex vivo* or *in vivo* tape-stripping data whereas, for EN, a good correlation was observed. No measurable permeation of either BMV or EN was detected in a 6-h *in vitro* skin penetration experiment.

**Conclusions:**

*In vitro* and *ex vivo* methods for topical bioequivalence determination can show correlation with *in vivo* outcomes. However, these surrogates have understandable limitations. A “one-size-fits-all” approach for topical bioequivalence evaluation may not always be successful, therefore, and the judicious use of complementary methods may prove a more effective and reliable strategy.

## INTRODUCTION

There is a pressing need to develop appropriate methods, as alternatives to clinical endpoint studies, to determine the bioequivalence of topical dermatological products (1). In general, regulatory agencies may accept different types of evidence to establish bioequivalence based upon how complex the dosage form is, and how similar formulations are to each other; for example, if solution formulations with the same amount of active ingredient contain the same inactive ingredients in the same amounts, then the risk of inequivalence may be considered to be inherently low. However, for semi-solid formulations that differ in excipient composition or dosage form (gel *versus* cream, for instance), amongst which the partitioning and/or diffusion of the active ingredient into and across the skin may be altered (2), it is imperative that surrogate *in vitro*, *ex vivo* and/or *in vivo* methods be optimized and validated to ensure that an effective and reliable determination of bio(in)equivalence be obtained.

The provision of less expensive medicines is the obvious driving force to identify procedures to facilitate the commercialization of bioequivalent, generic drug products (3, 4). With respect to oral delivery, the accepted approach is relatively straightforward and is principally based on matching blood level profiles (rate and extent of absorption) (5). For topical drug products other than the corticosteroids, a clinical trial is essentially and typically the only route for approval of a generic product or for replacement of an already approved dermatological product that has appreciable compositional changes (3). But, comparative clinical trials are relatively insensitive, time-consuming and costly; to gain the adequate statistical power needed to clearly evaluate bioequivalence may require a large number (*i.e.*, hundreds) of subjects (6).

There is an imperative, therefore, to validate one or more assessment approaches that might replace clinical efficacy studies to demonstrate bioequivalence (BE). The principal contenders for the determination of topical bioavailability (BA) and BE are summarized in Table I and may be separated into *in vitro* and *in vivo* approaches. The table identifies those methods, which have not yet received official sanction from the U.S. Food & Drug Administration as independent means with which to evaluate topical BA/BE, and others that have each, to some extent, been employed to compare different topical drug products (7).

**Table I:** Accepted and Investigational Methods for Assessing Topical Drug Product Bioavailability/Bioequivalence

Methods for topical bioavailability/bioequivalence		Currently accepted
*In vitro* approaches	Release tests (model membranes)	Yes
	Skin penetration experiments	No
*In vivo* approaches	Clinical trials	Yes
	Pharmacokinetics (blood/plasma levels)	Yes
	Pharmacodynamics (*e.g.*, vasoconstriction assay)	Yes
	Stratum corneum tape-stripping	No
	Dermal microdialysis	No

In this study, alternative methods to evaluate topical BE are considered for formulations of a corticosteroid, betamethasone valerate (BMV), and of an anti-fungal drug, econazole nitrate (EN), which have previously been examined in *in vivo* stratum corneum tape-stripping experiments in human volunteers (8, 9). For BMV, the formulations were prepared extemporaneously and were clearly inequivalent to one another when compared with the accepted vasoconstriction assay (8); the stratum corneum tape-stripping results were consistent with this finding. In the case of EN, the tape-stripping data confirmed the results of clinical trials that found the three creams examined to be bioequivalent. Here, the formulations of the two drugs are first subjected to *in vitro* release testing using model membranes, before being compared in an *ex vivo* tape-stripping protocol using porcine skin samples. A limited, but ultimately uninformative, *in vitro* skin penetration test (again using excised porcine skin) was also undertaken.

## MATERIALS & METHODS

### Formulations

Two betamethasone valerate (BMV, Sigma-Aldrich, Gillingham, UK) formulations were prepared, exactly as previously described (8): (a) dissolved in medium chain triglycerides (MCT) (Mygliol 812 N, Synopharm, Barsbüttel, Germany), and (b) in the microemulsion Mikro 100® (ME) (Sebapharma, Boppard, Germany). The vehicles were thickened into semi-solid gels with 6% (*w/w*) Aerosil® 200 (Sigma-Aldrich). The BMV concentration in each of the two formulations was adjusted to 80% of the drug’s solubility (9.3 and 1.7 mg mL^−1^ for ME and MCT, respectively), *i.e.*, to provide equivalent thermodynamic activity (8).

Similar to an earlier, detailed human *in vivo* tape-stripping study (9), three, commercially available econazole nitrate (EN) formulations (1% *w/v*) were considered: the reference listed product, Fougera® (E.Fougera & Co., Melville, NY), and two generic creams (listed as AB in the FDA Orange Book (10)) from Perrigo (Bronx, NY) and Taro (Hawthorne, NY).

#### *In Vitro* Release Test (IVRT)

BMV and EN transport from the various formulations was measured across either cellulose membranes (both hydrophilic, lot R2SA21096, and hydrophobic, lot R6AN36175, pore size 0.45 μm, from Whatman, Ltd., Little Chalfont, UK), or a non-porous silicone membrane (75 μm thickness, Dow Corning 7-4107, Auburn, MI). The membranes were soaked in phosphate-buffered saline (pH 7.4), containing 0.5% polyethylene glycol hexadecyl ether (Brij 58®, Sigma-Aldrich) for 0.5 h before mounting in standard Franz diffusion cells. The same solution as that used to pre-soak the membranes also provided the receptor phase (volume = 7.4 mL) and was chosen to ensure adequate drug solubility and the maintenance of sink conditions during the experiment. The jacketed diffusion cells were maintained at 32°C using a circulating water bath. Post-application of the BMV and EN formulations (221 and 4.5 mg/cm^2^, respectively (8, 9)), which were evenly spread over the membrane surface (2 cm^2^) facing the occluded donor compartment of the Franz diffusion cell, samples of the receptor phase (0.5 to 2 mL) were withdrawn at 0.25, 0.5, 0.75, 1, 2, 3, 4, 5 and 6 h for BMV, and at 0.5, 1, 2, 3, 4, 5 and 6 h for EN, and replaced with fresh receptor solution. The cumulative amount of drug released from each formulation as a function of time was assayed by high performance liquid chromatography using previously described methods (8, 9).

#### ***In Vitro*** Skin Penetration and ***ex Vivo*** Tape-Stripping Experiments

For the *in vitro* permeation test (IVPT) using excised porcine skin in Franz diffusion cells, the tested formulations were applied as in the IVRT experiments (221 and 4.5 mg/cm^2^ for BMV and EN formulations respectively, both occluded). The skin was sourced from a local abattoir, dermatomed (Zimmer dermatome, Dover, DE) to a nominal thickness of about 750 μm and then frozen at −20°C. Before use, the tissue was slowly thawed and mounted in the diffusion cell. The receptor medium was 7.4 mL of phosphate-buffered saline (pH 7.4) containing 0.5% w/v Brij 58®. Again, the jacketed diffusion cells were maintained at 32°C using a circulating water bath. The formulations were applied for 6 h (mimicking the earlier *in vivo* study design (8, 9)) at the end of which the cell was dismantled and the entire receptor phase contents were reserved for analysis of permeated drug. For BMV, the skin surface was cleaned of residual formulation either (a) by wiping with a dry paper towel, or (b) with this dry wipe *procedure plus* the use of two successive 70% *v/v* isopropyl alcohol swabs (Seton Healthcare, Oldham, UK). For EN, the skin surface cleaning procedure used only alcohol swabs as reported previously (9).

Subsequently, for both drugs, the skin was securely pinned to a polystyrene board and the central area was delimited with a template, the area of which equaled that exposed to the formulation. The stratum corneum (SC) at this site was then sequentially removed by adhesive tape-stripping (Scotch Book Tape, 3 M, St. Paul, MN for BMV, Shurtape J-LAR®, Avon, OH for EN) in accord with published procedures (2, 11). Concomitant measurements of transepidermal water loss (TEWL), made before and throughout the tape-stripping process, indicated that most, if not all, of the SC was removed (by which point TEWL had attained a value of 100 g/m^2^/h or more); the number of tape-strips required to do so was between 8 and 30. The adhesive tapes were weighed on a sensitive balance (Sartorius Microbalance SE-2 F, precision 0.1 μg; Sartorius AG, Göttingen, Germany) before and after skin stripping so that the mass of SC removed could be determined. As explained elsewhere (12–14), this information together with the corresponding change in TEWL as a function of the increasing quantity of SC removed allows the thickness of this barrier layer to be simply determined. The amount of drug removed on each tape-strip was then determined by extracting the drug from the adhesive by shaking overnight with an appropriate volume (in both cases 1 mL) of a suitable solvent: 40:60 *v/v* acetonitrile:water for BMV, pure methanol for EN. To optimize sensitivity, tape-strips from the deeper SC were usually analysed in groups of up to 4.

In a separate series of experiments with EN, once the skin surface had been cleansed of residual formulation at 6 h, the tissue was placed in an oven (maintained at 32°C; with the dermal side of the skin fully hydrated). After a further 17 h, the SC tape-stripping procedure was carried out exactly as described above. The objective of this component of the work was to mimic the ‘clearance’ period of the earlier human *in vivo* study (9).

### Data Analysis

#### IVRT

The results were presented as cumulative drug release as a function of time, and the behaviour of the different formulations compared. The most appropriate function describing the release profile (*e.g.*, linear, t^1/2^ kinetics) was assessed.

#### ***Ex Vivo*** Tape-Stripping

No measurable permeation of either BMV or EN into the diffusion cell receptor chamber was detectable in 6 h, obviating any need to interpret such data. For BMV, the drug concentration profile (C as a function of depth position x) across the SC after the 6-h uptake was fitted to the solution of Fick’s 2nd law of diffusion for constant vehicle concentration (C_veh_) at the surface (x = 0) of an initially drug-free SC:

$$ C=K\cdot {C}_{veh}\left\{\left(1-x/L\right)-\frac{2}{\pi }{\displaystyle \sum_{n=1}^{\infty}\frac{ \sin \left(n\pi x/L\right)}{n}}\cdot \exp \left(-\left(D/{L}^2\right){n}^2{\pi}^2t\right)\right\} $$

to derive values of the SC-vehicle partition coefficient (K) and the ratio of the drug’s SC diffusivity to the SC thickness squared (D/L^2^) as explained in earlier work (11, 15). Additionally, the permeability coefficient across the SC (k_p_) and the steady-state flux (J_ss_) were estimated using the independent assessment of SC thickness.

In the case of EN, a more straightforward analysis of the results was undertaken, mirroring the approach adopted in the published *in vivo* tape-stripping study performed in human volunteers (9). Here, the uptake and clearance of the drug were determined from the total drug amounts recovered from the SC tape-strips collected either immediately or 17 h after cleaning.

### Statistics

As the goal of this research was not to establish bio(in)equivalence between the different formulations of the two drugs considered, the number of replicates employed in the *in vitro* and *ex vivo* parts of the study were not based on rigorous power calculations. Rather, the values of “n” employed were selected to match those which had been used in the previous *in vivo* experiments (*n* = 6 for BMV and *n* = 14 for EN) (8, 9).

Statistical analysis involved two-tailed Student’s t-tests and one- and two-way analyses of variance (ANOVA) followed by Bonferroni’s test; p-values less than 0.05 were considered statistically significant.

## RESULTS

### ***In Vitro*** Release Tests

IVRT with the BMV formulations revealed that no measurable amount of the drug transported into the receptor phase across the porous hydrophobic or silicone membranes. BMV release was observed across the hydrophilic membrane, however. From the microemulsion gel (ME), 1430 (±161) μg cm^−2^ was released in 6 h, while the corresponding amount from the medium chain triglyceride formulation (MCT) was 7.7 (±0.8) μg cm^−2^. The large difference in the two quantities may have been caused by the simultaneous diffusion of surfactant from the ME gel facilitating solubilisation of BMV in the receptor phase. For both formulations, drug release was described by a typical square root of time dependence.

**Fig. 1:**
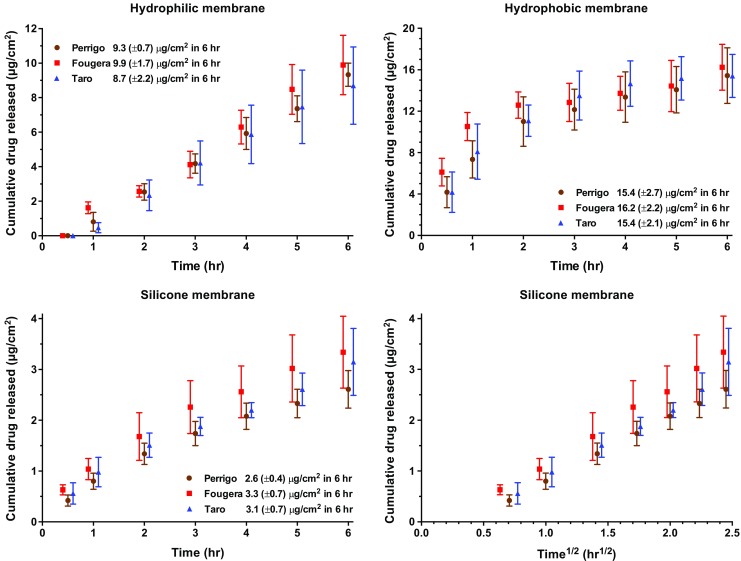
EN release (mean ± SD; *n* = 6) from three commercially available creams across three artificial membranes. Data have been staggered on the time axis for clarity and the square root of time transformation of the results from the silicone membrane is illustrated in the lower right-hand panel of the figure.

Release of EN from all three formulations across each of the three membranes used was detected (Fig. 1). While the cumulative amounts released in 6 h were significantly different depending on the membrane used (ANOVA followed by post-hoc test), within each membrane there was no significant difference in drug release from the three formulations.

### ***Ex Vivo*** Skin Penetration

At the end of the 6-h experiment, no BMV was found in the receptor solution demonstrating its inability to cross the skin regardless of the vehicle used within this short time-frame. The same was true for EN, a finding consistent with the earlier *in vivo* tape-stripping investigation, the results of which indicated a lag-time of ~13 h (9).

**Fig. 2:**
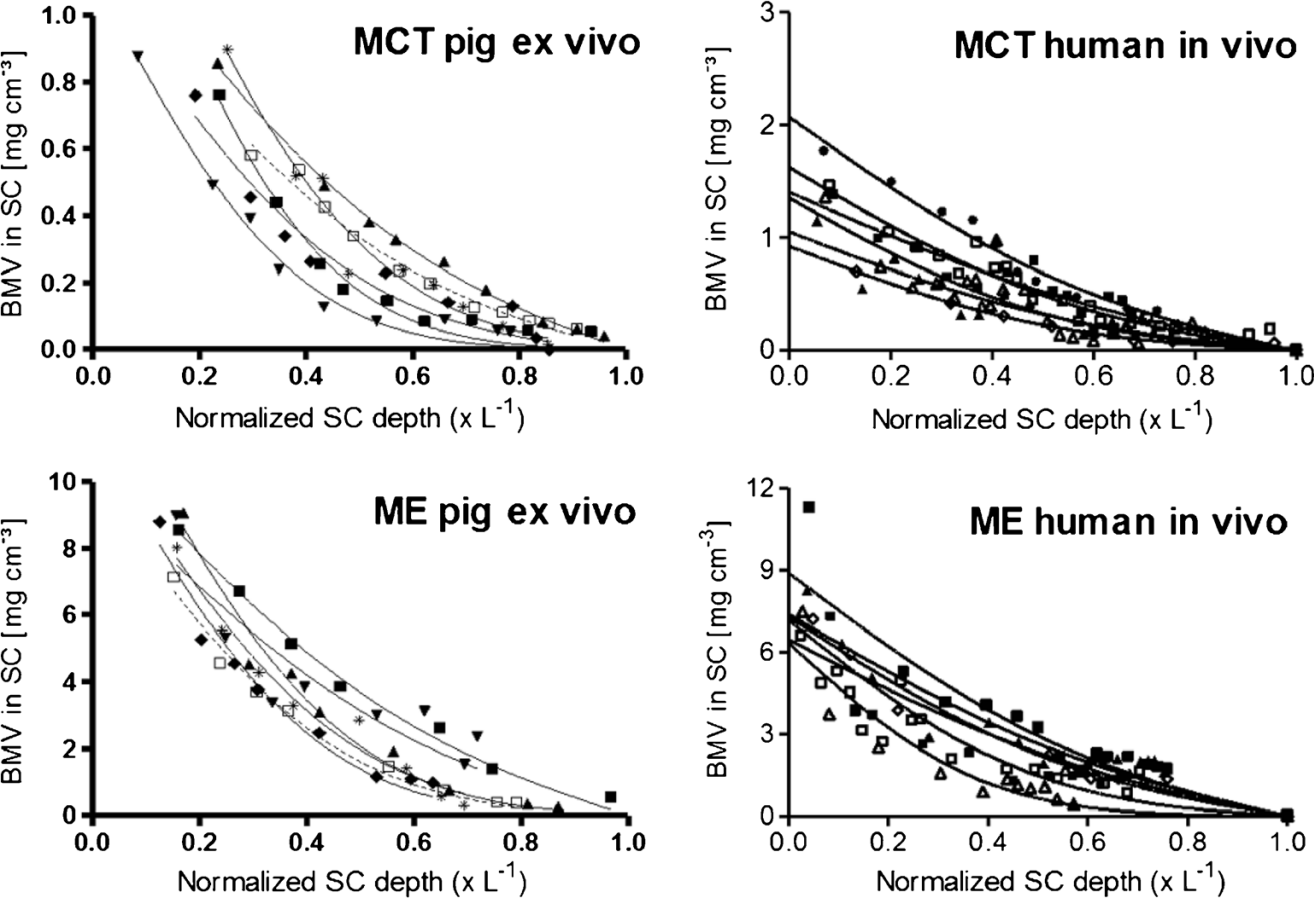
BMV concentration profiles (*n* = 6) across porcine SC *ex vivo* (left panels, this work) and across human SC *in vivo* (7) (*right panels*) following a 6-h application of the drug in either a microemulsion (ME) or a medium chain triglyceride formulation (MCT).

Figure 2 (left panels) presents BMV concentration profiles as a function of position within the SC, determined from the *ex vivo* tape-stripping experiments following the 6-h treatment with the gelled medium chain triglyceride (MCT) and microemulsion (ME) gel formulations; the skin surface was wiped clean with dry tissue in these experiments. The data are compared to the corresponding results (*right panels*) redrawn from the published *in vivo* study conducted on human volunteers (8).

**Fig. 3:**
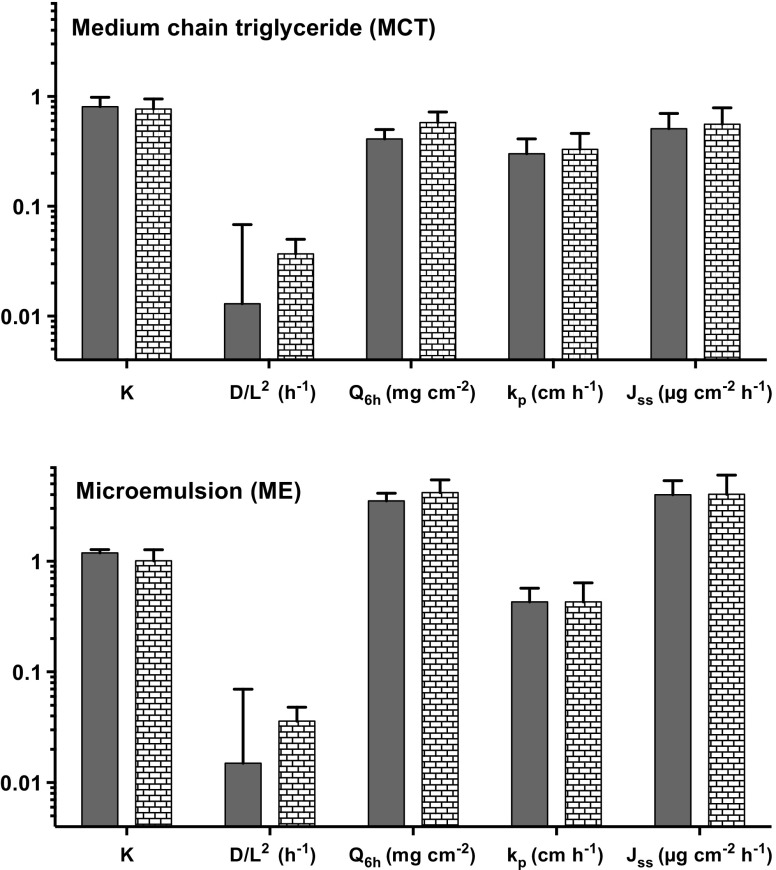
Derived values (*n* = 6, mean ± SD) of BMV SC-vehicle partition coefficients (K), diffusivity parameters (D/L^2^), permeability coefficients (k_p_) and apparent steady-state fluxes (J_ss_), as well as the total drug quantities taken up into the SC in 6 h (Q_6h_), following delivery from MCT and ME formulations. The filled bars are results derived from the *ex vivo* porcine skin experiments reported here; the stippled bars represent data from a published *in vivo* study using the same formulations and methodology (8).

Partitioning and apparent diffusion parameters derived as described above (15) are summarized in Fig. 3 along with the total amounts of drug taken up into the SC at 6 h, and estimated values of the permeability coefficients and apparent steady-state fluxes. These results are again compared with those reported from the earlier *in vivo* experiments (8, 9). For both formulations, there was excellent agreement (and no significant difference) between the *ex vivo*-derived parameters and those from the *in vivo* human study; equally, as observed from the tape-stripping experiments in human volunteers, the uptake of BMV into the SC and the apparent steady-state flux of the drug, were almost an order of magnitude greater from the microemulsion compared to the MCT formulation (8.7-fold *ex vivo versus* 7.2-fold *in vivo*).

When the *ex vivo* experiments were repeated with the skin being cleaned more rigorously with isopropyl alcohol swabs, the Q_6h_ values for both formulations were reduced by about 50% (data not shown), confirming that this approach is a more robust method with which to remove residual formulation (16).

**Fig. 4:**
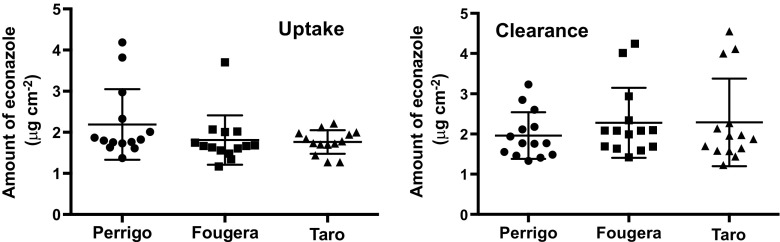
Total amounts of econazole recovered in the SC, following uptake and clearance periods of 6 and 17 h, respectively, in *ex vivo* tape-stripping experiments (*n* = 14) with three commercially available EN creams.

The *ex vivo* tape-stripping experiments with econazole nitrate (EN) were undertaken using the same protocol as that used in the published *in vivo*, human study (9). The uptake and clearance of the drug from the SC were determined in an identical number of replicates, taking care to thoroughly cleanse the skin surface after the 6-h exposure to the three creams and to ensure that essentially all of the SC was removed in the tape-stripping procedure. Figure 4 reports the amounts of EN recovered from the SC after the 6-h uptake and subsequent 17-h clearance periods.

Analysis of variance of the results for both uptake and clearance shows that there was no significant difference between the three formulations considered. Also noteworthy is that for each EN cream, there was no significant difference between the drug amounts recovered in the SC after uptake and clearance periods. The mean values, and the upper and lower 90% confidence intervals (C.I.) on the data obtained, are collected in Table II.

**Table II:** Average Quantities (*n* = 14) of Econazole in the SC After Uptake and Clearance Periods Following Application of Three Drug Products, Together with the Corresponding Upper and lower 90% Confidence intervals (C.I.)

Quantity of EN (μg cm^−2^)		Drug product		
		Perrigo	Fougera	Taro
Uptake	Mean	2.19	1.81	1.77
	Lower 90% C.I.	1.61	1.17	1.44
	Upper 90% C.I.	4.18	3.70	2.22
Clearance	Mean	1.96	2.28	2.80
	Lower 90% C.I.	1.48	1.42	1.44
	Upper 90% C.I.	3.23	4.24	4.88

As the uptake and clearance values were indistinguishable for each of the three creams tested, equivalence between the products was assessed using the combined results and the ratios of [uptake + clearance] for the mean values of the two test formulations (Perrigo and Taro) to that of the reference (Fougera). The calculations were undertaken using both the raw data (Fig. 4) and the log transformed results. The outcome was essentially identical, in terms of the ratios falling within the conventional range of 0.8 to 1.25 (2, 17), and is summarized in Table III.

**Table III:** Simulated Bioequivalence Assessment, Based on Combined [uptake + clearance] Data (*n* = 28), Between the Three EN Creams with Fougera Serving as the ‘Reference product’ and Perrigo and Taro as the ‘tests’

Bioequivalence assessment	Perrigo *vs.* Fougera	Taro *vs.* Fougera
Average ratio (test/reference)	1.02	0.99
Lower 90% C.I.	0.89	0.87
Upper 90% C.I.	1.17	1.12

## DISCUSSION

The IVRT results show distinct behaviour between the two drugs considered. On the one hand, EN release from the three products tested was easily measurable across the three different artificial membranes used. In addition, the release characteristics for the different formulations were the same across each individual membrane. However, the profiles did not overlap quantitatively when comparing the data obtained from the different membranes, and the shape of the profile across the hydrophilic cellulose barrier was distinct from that across the two hydrophobic membranes (Fig. 1). For BMV, over a period of 6 h, drug release was not measurable through the two hydrophobic membranes, presumably reflecting the high solubility of the drug in these barriers. In contrast, release through hydrophilic cellulose was detectable and proceeded with a classic square-root-of-time dependence, which distinguished between the two formulations tested.

The message from these experiments should be clear and has been articulated before (18). Specifically, while IVRT can provide useful quality control information about the consistency of (for example) different production batches, it is unwise to predict drug bioavailability *in vivo*, either relative or absolute, from these measurements alone. The EN data show that the amounts released differ depending upon the membrane employed and that the quantities ‘delivered’ across the membranes can be substantially higher than even those amounts which only enter the SC in the same period. Although the apparent equivalence shown by IVRT of the three EN products is mirrored in both *in vivo* and *ex vivo* tape-stripping studies (and is indeed consistent with clinical performance too), any deduced correlation should be carefully considered in light of the results for BMV. For this drug, in two cases, IVRT shows no permeation of drug whatsoever. It follows, therefore, that not only is it very unlikely that a single artificial membrane can be used to standardize the IVRT approach for all drugs, but also, even if one did, that it would be capable of mimicking any formulation effects (*e.g.*, the action of an excipient which is a penetration enhancer) on real skin.

The *ex vivo* SC tape-stripping experiments with BMV showed extremely good qualitative and quantitative agreement with previously published (8) human *in vivo* studies (Figs 2 and 3). The results demonstrate that carefully conducted studies on excised skin (and, in this instance, excised skin from a recognized and generally-considered acceptable model for the human barrier (19, 20)) can be usefully predictive of the *in vivo* situation, as has been intimated before (21). While it may be unlikely that this strategy would eventually evolve into any sort of regulatory guidance, the availability of an alternative, *ex vivo* approach may be attractive in formulation development and optimisation.

The results from the EN *ex vivo* tape-stripping study were mixed from the standpoint of correlation with the previously published *in vivo* human data (9). On the positive side, the uptake of the drug into the SC of excised porcine skin over 6 h correlated completely with the data in man and (correctly) demonstrated the equivalence between the three drug products tested (Tables II and III). In contrast, while the results from the clearance part of the study were self-consistent in that they also indicated the equivalence of the formulations (Fig. 4), the data diverged, however, from the earlier *in vivo* observations. *In vivo*, there was about a 30% reduction in the SC level of econazole during the clearance phase but, *ex vivo*, there was no decrease at all. The most likely and obvious explanation for this observation is that excised skin lacks a functioning microcirculation and fails to provide, as a result, the sink conditions necessary to clear a very lipophilic drug like econazole. This active moiety prefers to remain in the SC, therefore, and does not deplete significantly over the 17-h period subsequent to the removal of residual formulation. This implies that an *ex vivo* tape-stripping approach to assess topical bioequivalence may not routinely furnish information on the elimination aspect of ‘skin pharmacokinetics’, especially for drugs with high log P values. However, this does not mean that such experiments are without value; on the contrary, data on the uptake phase are extremely useful for optimising the design of an *in vivo* experiment and for providing valuable insight into the performance of prototypical formulations being considered for clinical evaluation. That having been said, suitable modifications to the protocol used here may permit this approach to also shed light on the clearance process; for example, maintaining the skin in contact with a receptor chamber of large volume, or with a flow-through option, and using thinner sections of excised skin are strategies worthy of investigation.

In conclusion, the results of this investigation confirm that techniques, such as IVRT and SC tape-stripping, are robust approaches with which to characterise aspects of topical drug product performance that contribute to the active pharmaceutical ingredient’s ultimate bioavailability in the skin. However, each of the methods used here have limitations that have been articulated above: IVRT can address features of the formulation’s quality, but cannot report on the manner in which the product will interact with the skin; *ex vivo* tape-stripping permits good prediction of drug uptake into the SC *in vivo* but, with respect to the determination of drug clearance, careful attention needs to be paid to the optimisation of the experimental design. Because of the brevity of the experiments performed, no useful information on drug permeation through excised skin was obtained; nevertheless, it is clear that classic *in vitro* penetration experiments also have an important role to play in the armoury of tools available for the assessment of topical bioavailability.

## ABBREVIATIONS

ANOVA: Analysis of variance
BA: Bioavailability
BE: Bioequivalence
BMV: Betamethasone valerate
C.I.: Confidence interval
C_veh_: Drug concentration in vehicle
EN: Econazole nitrate
IVPT: *In vitro* penetration test
IVRT: *In vitro* release test
J_ss_: Steady-state flux
K: Stratum corneum – vehicle partition coefficient
k_p_: Permeability coefficient
log P: Logarithm of the octanol-water partition coefficient
MCT: Medium chain triglyceride
ME: Microemulsion
Q_6h_: Quantity absorbed in 6 h
SC: Stratum corneum
TEWL: Transepidermal water loss
